# Radio-lncRNAs: Biological Function and Potential Use as Biomarkers for Personalized Oncology

**DOI:** 10.3390/jpm12101605

**Published:** 2022-09-29

**Authors:** Joanna Kozłowska-Masłoń, Kacper Guglas, Anna Paszkowska, Tomasz Kolenda, Marta Podralska, Anna Teresiak, Renata Bliźniak, Katarzyna Lamperska

**Affiliations:** 1 Laboratory of Cancer Genetics, Greater Poland Cancer Center, Garbary Street 15, 61-866 Poznan, Poland; 2 Research and Implementation Unit, Greater Poland Cancer Center, Garbary Street 15, 61-866 Poznan, Poland; 3 Institute of Human Biology and Evolution, Faculty of Biology, Adam Mickiewicz University, Uniwersytetu Poznańskiego 6, 61-614 Poznan, Poland; 4 Postgraduate School of Molecular Medicine, Medical University of Warsaw, Zwirki and Wigury Street 61, 02-091 Warsaw, Poland; 5 Faculty of Biology, Adam Mickiewicz University, Umultowska 89, 61-614 Poznan, Poland; 6 Institute of Human Genetics, Polish Academy of Sciences, Strzeszynska 32, 60-479 Poznan, Poland

**Keywords:** radiotherapy, personalized medicine, lncRNA, ncRNA, biomarker, therapeutic target, irradiation, cancer

## Abstract

Long non-coding RNAs (lncRNAs) consist of at least 200 nucleotides. Although these molecules do not code proteins, they carry many regulatory functions in normal cells, as well as in cancer cells. For instance, many of these molecules have been previously correlated with tumorigenesis of different cancers and their reaction to various stress factors, such as radiotherapy, chemotherapy, or reactive oxygen species (ROS). The lncRNAs are associated not only with dysregulation in cancers after applied treatment but also with beneficial effects that may be achieved by modulating their expression, often significantly enhancing the patients’ outcomes. A multitude of these molecules was previously considered as potential biomarkers of tumor development, progression, or cells’ response to radio- or chemotherapy. Irradiation, which is often used in treating numerous cancer types, is not always sufficient due to cells gaining resistance in multiple ways. In this review, studies considering lncRNAs and their reaction to radiotherapy were examined. These molecules were divided regarding their role in specific processes strictly related to irradiation, and their influence on this type of treatment was explained, showing how vast an impact they have on IR-supported combat with the disease. This review aims to shed some light on potential future lncRNA-based biomarkers and therapeutic targets.

## 1. lncRNAs Are New Players in Radiogenomics

It is well known that genetic background has a pivotal influence on the radioresistance of normal and cancer cells. Current research on radiosensitivity focuses on comprehensive genome analyses that take into account mutations in genes, as well as changes at the transcriptome and epigenome levels [1,2]. Unfortunately, to this day, it has not been possible to create specific mutation panels enabling the determination of clinical response to the applied radiotherapy. Radiogenomics is “the study of genomic changes that underlie the radioresponse of normal and tumor tissues” and is likely to enable a breakthrough in personalized radiotherapy [3]. It should be emphasized that the radiogenomic research conducted so far has focused on mRNA transcripts, protein-coding genes, and epigenetic elements—miRNAs [1,4,5]. However, the epigenome is constructed by many different types of RNA molecules, which interact and together create complex networks consisting of mRNAs, miRNAs, and other non-coding RNAs (ncRNAs) [6,7].

ncRNAs can be divided into two groups: constitutive RNAs such as transfer RNA (tRNA), ribosomal RNA (rRNA), small-nuclear RNA (snRNA), and small-nucleolar RNA (snoRNA); and regulatory RNAs including small interfering RNA (siRNA), piwi-interacting RNA (piRNA), microRNA (miRNA), natural antisense transcripts (NAT), circular RNA (circRNA), and long non-coding RNA (lncRNA) [1,2,3]. It should be mentioned that this list is incomplete, and periodically new dependencies or even elements of the epigenome are discovered. This is possible due to the development of databases, analytical techniques, bioinformatics, and the use of artificial intelligence (AI) [3,8,9,10]. As a result, the so-called genetic noise, the unknown or omitted functional RNA molecules, have been discovered and now are known as lncRNAs. In addition, they are also partly classified as pseudogenes [11,12]. Although these molecules do not encode proteins, over time they have become valuable and significant elements of cell biology. Their sequence consists of more than 200 nucleotides and very often contains components typical for mRNA, namely poly-A tails and regulatory sequences, such as those for miRNAs. The activity of lncRNAs is characterized not only by interaction with proteins and other RNAs but also by regulation of transcription and expression of genes through changes to the chromatin structure. Genes encoding the mentioned molecules can occupy intergenic or intragenic positions, of which a specific intragenic gene may be located in the intron, transcriptional enhancer elements, promoter, or 3′UTR flanking regions [11,13]. 

The process of lncRNA biogenesis is regulated by RNA polymerase II and is similar to mRNA formation. After transcription, the cap structure is attached to the molecule; then it undergoes polyadenylation and splicing [14]. lncRNAs appear to be pivotal in functions related to the regulation of gene transcription in the nucleus or subsequent post-transcriptional modifications in the cytoplasm. However, due to their location in the genome, it is difficult to fully understand the function of lncRNAs. It should be noted that more than 50% of lncRNAs are long intergenic non-coding RNAs (lincRNAs) [15]. The direction of transcription and the distance at which the transcript responsible for encoding the protein is located in relation to the lincRNA has made it possible to divide this group of RNAs into four classes: (i) located on the same strand, (ii) convergent, (iii) divergent, and (iv) isolated, which are located at least 50 kb from the nearest gene encoding the protein [15]. The main challenges in understanding the mechanisms of action of lncRNAs stem from the varying levels of expression depending on tissue localization [16], the frequent heterogeneity of isoforms, and the numerous repeats in regions of transcription initiation [17]. Moreover, since it has been shown that lncRNA activity changes with different levels of their expression, its specificity in various cell types and tissues should be assessed [18]. As it turned out, abnormalities in activity or biogenesis mechanisms of these molecules can appear in states of pathological conditions and indicate cancer progression by affecting a multitude of transcription factors along with the structure of the chromatin [19]. It has been demonstrated that the aberration of not only coding RNAs but also non-coding RNAs plays a crucial role in cancer biology. Although the function and activity of lncRNAs are still under investigation, in the future they may become crucial tools for predicting the development and possible treatment of cancer [19].

It should be noted that changes in the epigenetic system can be caused by even small doses of irradiation (IR). Li et al. indicated that hepatocarcinogenesis can be caused by changes in the expression of HULC lncRNA, which is an IR-induced molecule. The abovementioned RNA regulates the neighboring gene, CDKN1, by complementary base pairing, and in turn promotes cell cycle progression [20]. Additionally, apart from local, tissue-based changes in lncRNA expression, IR also causes a systematic, whole organism response. Aryankalayil et al. observed that after mice whole-body IR, using different doses of X-rays, expression levels of different lncRNAs were dramatically changed in the whole blood. The radiation-induced RNAs were connected with DNA damage response and the immune system. Moreover, it has been proven that two lncRNAs, Gm14005 (Morrbid) and Tmevpg1, could be used as potential biomarkers after radiation exposition [21]. 

Studies indicate that epigenetic mechanisms are crucial for the IR-induced changes in reactive oxygen species (ROS) generation, as well as that oxidative stress itself can modulate epigenetics in the cells exposed to radiation [22]. lncRNAs, due to their vast influence on cellular processes, including DNA damage repair, cell cycle arrest, apoptosis, senescence, maintaining cancer stem cell populations, the epithelial-to-mesenchymal transition (EMT) process, and autophagy, seem to play an important role in the response to IR and are new players in radiobiological research [23].

In this review, the current knowledge about lncRNAs involved in radiobiological processes, as well as their use as potential biomarkers in oncology, especially in personalized radiotherapy, is discussed.

## 2. Involvement of lncRNAs in Radiobiological Processes

**Figure 1:**
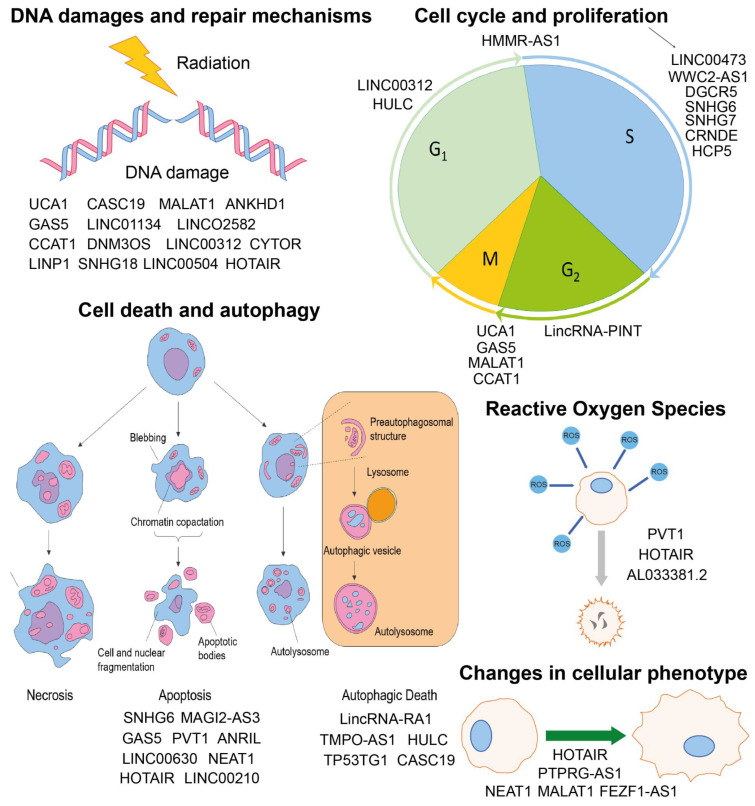
lncRNAs in different processes associated with radioresponse, including: DNA damages and repair mechanism, cell cycle and proliferation, cell death and autophagy, regulation of reactive oxygen species (ROS), and changes in cellular phenotype.

lncRNAs are pivotal regulators of cell biology that have a significant impact on cancer development. However, these molecules also play a crucial role in adverse effects associated with radiotherapy. One such complication, radiation-induced intestinal fibrosis (RIF), is frequent in abdominal and pelvic tumor treatment. Zhou et al. determined that the lncRNA WWC2-AS1 is overexpressed in RIF compared to adjacent tissue, and its knockdown significantly inhibited proliferation, invasion, and migration of changed tissue, presumably through modulating the miR-16/FGF2 axis. A therapeutic strategy targeting this lncRNA could reduce RIF progression and lead to improvement in patients’ life quality [24]. Such discoveries emphasize the importance of RNA-based cancer research in the context of tumor biology and in the accompanying changes throughout the organism before, during, and after treatment; see Figure 1.

### 2.1. Cell Cycle and Proliferation

One of the lncRNAs that have a notable impact on the cancer cell cycle and proliferation is the small nucleolar RNA host gene 7 (SNHG7) lncRNA. Chen et al. observed that the SNHG7/miR-9-5p/DPP4 axis modulates tumor growth based on analyses of ^131^I-resistant thyroid carcinoma cell lines. The examined lncRNA acted as a molecular sponge sequestering miR-9-5p, which led to an increase in dipeptidyl-peptidase 4 (DPP4) expression levels resulting in activation of the PI3K/Akt signaling pathway. The authors emphasized the importance of this regulatory axis as a future therapeutic target [25]. Experiments by Guo et al. discovered that in esophageal cancer, lncRNA HCP5 modulates the Akt signaling pathway via regulating the miR-216a-3p/PDK1 axis. Its knockdown combined with irradiation led to suppression of proliferation, a decrease in AKT activation, and an increase in apoptosis rate, consequently improving radiosensitivity [26]. 

Small nucleolar RNA host gene 6 (SNHG6) is significantly overexpressed in breast cancer (BC) cells and tissues compared to normal cells. It positively correlates with laminin subunit gamma 1 (LAMC1) and negatively with miR-543. The downregulation of SNHG6 resulted in inhibition of cell migration and proliferation, impaired colony formation, enhanced apoptosis, and radiosensitivity of BC cells. Additionally, downregulation of this lncRNA also decreased protein levels of Snail and vimentin but increased E-cadherin, suggesting that its depletion suppressed EMT processes in BC cells. Interestingly, miR-543, which is negatively correlated with SNHG6, can change to the opposite of all of the above when overexpressed in BC cells. The knockdown of the abovementioned lncRNA also decreased levels of LAMC1, p-PI3K, and p-AKT in the PI3K/AKT pathway. All of the results above show how vast an impact SNHG6 in BC has on radiotherapy response [27].

Colorectal neoplasia differentially expressed (CRNDE) lncRNA is significantly upregulated in ovarian cancer cells and correlated with their radioresistance. CRNDE silencing resulted in enhanced radiosensitivity and inhibited clone formation and tumor growth in mice [28].

Another molecule influencing the Akt pathway activation is urothelial carcinoma-associated 1 (UCA1). A study by Fotouhi Ghiam et al. indicated that this molecule is significantly upregulated in prostate cancer (PCa), and its high level is associated with an unfavorable prognosis. Experimentally induced depletion of UCA1 in cell lines abrogated the aggressive phenotype and potentiated radiosensitivity via reducing proliferative capacity, leading to cell cycle arrest at the G2/M transition and inhibiting activation of the pro-survival Akt pathway. It was determined that this lncRNA has significant prognostic and therapeutic potential in PCa [29]. A different lncRNA linked with PCa irradiation (IR)-based treatment is GAS5. Yung et al. discovered that radiotherapy supported by α-Solanine administration caused upregulation of the said lncRNA and downregulation of miR-18a, which led to inhibition of cells’ proliferation ability and promotion of apoptosis via an increasing level of γ-H2AX [30]. The above interaction seems to be a fascinating new therapeutic approach, which could provide sensitization to radioresistant tumors.

The aforementioned GAS5 can also modulate G2/M arrest in malignant cells. Ma et al. discovered that it was downregulated in the BC cell line, and its level further decreased after IR. Moreover, they have proven that induced GAS5 overexpression assisted by radiotherapy suppressed cell proliferation, increased the DNA damage rate, and promoted activation of apoptosis, elevating Bax and caspase-3 expression levels. This pro-apoptotic response could be diminished by miR-21; however, it is negatively regulated by the studied lncRNA. Although the exact mechanism has not been fully described yet, the presented results imply that GAS5 plays a pivotal role in radiosensitization [31]. A different molecule regulating G2/M arrest is lncRNA MALAT1. Analysis carried out on high-risk human papillomavirus (HR-HPV)-positive cervical cancer (CC) cell lines showed that this molecule modulated radiotherapy response via sequestering miR-145. Depletion of MALAT1 resulted in an elevated ratio of G2/M checkpoint arrest and cell apoptosis, which induced radiosensitivity [32].

Colon cancer-associated transcript 1 (CCAT1) in NSCLC cells is upregulated, causing high radioresistance and a low apoptosis rate of lung cancer cells. On the other hand, downregulation of this lncRNA resulted in radiosensitivity improvement of NSCLC cells by mediating cell cycle arrest, increased apoptosis rate, and DNA damage. Downregulation of CCAT1 arrests cells at G2/M, which is believed to be the most radiosensitive moment for cells, and further promotes radiation-induced γH2AX expression, a DNA damage marker. Silencing CCAT1 may also block MAPK signaling pathways and decrease p-p38/p38, p-ERK/ERK, and p-JNK/JNK [33].

Li et al. carried out analyses indicating that lncRNA HMMR-AS1 is highly expressed in glioblastoma (GM) cell lines and stabilizes mRNA of HMMR oncogene via sense-antisense interference. Depletion of lncRNA leads to a reduction in HMMR level resulting in cell arrest at the G1/S checkpoint and consequently inhibits tumor growth, cell migration, and the mesenchymal phenotype. The described interaction might become a very interesting possible therapeutic strategy [34].

A study by Liu et al. identified the LINC00473/miR-497-5p/CDC25A axis, which modulates esophageal squamous cell carcinoma (ESCC) cell lines’ proliferative ability and response to IR. Overexpression of said lncRNA was linked with a more advanced T stage, lymph node metastasis, and less differentiated tumor tissue. LINC00473′s oncogenic function could be abrogated by induced overexpression of miR-497-5p, resulting in sensitization to radiotherapy [35]. Interestingly, Chen et al. implied that LINC00473 elevated SPIN1 expression by negatively regulating miR-374a-5p, resulting in the promotion of cell proliferation and radioresistance. Further studies showed that both LINC00473 and SPIN1 competed for miRNA, and their high expression levels were correlated with poor prognosis and weakened response to IR [36]. 

Tang et al. reported that DiGeorge syndrome critical region gene 5 (DGCR5) is a lncRNA associated with larynx squamous cell carcinoma (LSCC) progression and resistance to treatment. This molecule acts as a molecular sponge and negatively regulates miR-195. The authors proposed that both silencing of DGCR5 and inducing expression of miRNA have a beneficial impact on supporting effects of IR treatment [37].

### 2.2. Cell Death and Autophagy

Radiotherapy can induce the death of cancer cells in different pathways, such as apoptosis, pyroptosis, mitotic catastrophe, necrosis, or autophagy.

Liu et al. determined that lncRNA LINC00630 binds to EZH2 and subsequently negatively regulates the *BEX1* gene via enhancing its promoter methylation in colorectal cancer (CRC) cell lines. Epigenetic silencing of *BEX1* results in suppression of apoptosis, enhancement of cell viability, and strengthening of the radioresistance mechanisms. On the other hand, lncRNA silencing significantly improved the sensitivity of cancer cells after IR. Although the exact nature of this interaction has not been fully described yet, the LINC00630/EZH2/BEX1 axis seems to be a new fascinating direction of study [38]. A different lncRNA, whose knockdown might notably improve the efficacy of radiotherapy in CRC patients, is HOX transcript antisense RNA (HOTAIR). It has been proven that lncRNA acts as a molecular sponge for miR-93, reducing its level and leading to an increase of the ATG12 expression level. HOTAIR silencing resulted in a decrease in cell viability and survival rates, as well as a reduction of pro-apoptotic protein expression, which caused activation of apoptosis and an increase in levels of p62, cleaved caspase-3, and Bax in studied cell lines [39]. Contrary to the LINC000630 and HOTAIR, the effect of lincRNA-p21 overexpression in CRC enhances the beneficial effects of radiotherapy. Its level tends to rise after applied IR and reinforces radiosensitivity by potentiating cell apoptosis. Induced upregulation inhibited Wnt/β-catenin signaling and elevated the expression level of the pro-apoptotic gene—Noxa [40].

The ESCC-based study by Lin et al. indicated that simultaneous overexpression of lncRNA GAS5 and downregulation of miR-21 cause elevation of RECK expression levels, strengthening radiosensitivity after IR. The obtained effect seems to result from enhanced apoptosis. Moreover, levels of all three molecules were correlated with clinical features such as TNM staging, degree of differentiation, lymph node metastasis, and distant metastasis [41].

In nasopharyngeal carcinoma (NPC), lncRNA PVT1 has been associated with unfavorable prognosis and radioresistance promotion. He et al. proved that its silencing sensitized cell lines to the applied treatment leading to inhibition of proliferation and induction of apoptosis. The depletion of PVT1 resulted in a reduction of ATM, Chk2, and p53 phosphorylation levels, as well as the activation of pro-apoptotic proteins. The above underlines the prognostic and therapeutic potential of the said molecule [42].

Membrane-associated guanylate kinase, WW, and PDZ domain containing 2 antisense RNA 3 (MAGI2-AS3) lncRNA is poorly expressed in ESCC. It is known to negatively correlate with homeobox protein Hox-B7 (HOXB7), which is overexpressed in ESCC and contributes to cancers’ radioresistance. MAGI2-AS3 downregulates HOXB7 via histone methyletransferase EZH2 to initiate H3K27me3, which is required for the EZH2-mediated repression of various genes that are vital for carcinogenesis and tumor development. H3K27me3 has the ability to suppress HOXB7 and its functions, such as developing radioresistance in ESCC cells. MAGI2-AS3 overexpression inhibits ESCC cells’ proliferation and resistance to radiotherapy as well as promotes cell apoptosis by downregulating HOXB7. In conclusion, MAGI2-AS3 overexpression enhances the radiosensitivity of ESCC cells to radiotherapy through the downregulation of HOXB7 via EZH2 [43].

The lncRNA antisense non-coding RNA in the INK4 locus (ANRIL), the antisense RNA1 of CDKN2B, is highly expressed in colon cancer tissues and cell lines, inhibiting apoptosis and causing resistance to radiotherapy. The upregulation of ANRIL inhibited chitooligosaccharide (COS)-induced radiosensitivity in colon cancer cells by targeting miR-181a-5p. Knockdown of said lncRNA or upregulating miR-181a-5p, which is negatively correlated with ANRIL, may change that effect on colon cancer cells, resulting in radiosensitivity enhancement [44].

In osteosarcoma cells and tissues, LINC00210 is significantly elevated and correlated with enhanced radioresistance. On the other hand, LINC00210 knockdown inhibits colony formation ability, decreases cell viability, and significantly induces apoptosis by inhibiting levels of cyclin D1 and Bcl-2, as well as overexpressing p21 and Bax. The abovementioned lncRNA is negatively correlated with miR-342-3p, which may reverse all effects of LINC00210 up- or downregulation. Additionally, LINC00210 is positively correlated with GFRA1, which when downregulated, also increases the radiosensitivity of osteosarcoma cells [45].

A different kind of IR-induced programmed cell death is pyroptosis—an inflammatory process that reduces the proliferation and invasiveness of cancer cells. It has been proven that it is regulated by lncRNA NEAT1 in CRC cell lines through the miR-448/GSDME axis. The expression level of the studied lncRNA was upregulated in tumor tissue and increased further after IR in a time-dependent manner, promoting GSDME-mediated pyroptosis. Activation of this type of cell death leads to an increase in the radioresistance of tumor cells [46]. 

The process of IR-promoted autophagy could protect in detrimental conditions, provided that it is not extensive, then it could activate apoptosis. Jiang et al. conducted an in silico analysis of autophagy-related lncRNAs in lung adenocarcinoma (LUAD). They discovered molecular signatures consisting of lncRNAs TMPO-AS1 and BIRC5, which were both overexpressed in patients’ samples. These molecules’ co-expression has been correlated with the advanced stage of the disease and unfavorable outcomes, suggesting the emergence of interesting future therapeutic targets [47]. A study by Gao et al. showed that lncRNA TP53TG1 is upregulated in glioma cell lines, and its expression level tends to rise after radiation. Moreover, its high levels potentiated tumor progression and radioresistance via autophagy activation. The authors indicated that lncRNA depletion reduced tumor growth and promoted apoptosis through the TP53TG1/miR-524-5p/RAB5A axis, improving radiotherapy effectiveness [48]. Another lncRNA involved in the activation of autophagy processes in glioma is linc-RA1. Zheng et al. proved that lincRNA stabilized the level of H2B K120 monoubiquitination (H2Bub1) via binding with H2B, subsequently reducing the interaction between H2Bub1 and ubiquitin-specific protease 44 (USP44). The described axis leads to autophagy inhibition and radioresistance progression [49]. The above underlines the still ambiguous role of autophagy in the process of cancer treatment.

Hepatocellular carcinoma upregulated long non-coding RNA (HULC) is significantly overexpressed in PCa cells. Its downregulation causes notably higher cell radiosensitivity. HULC downregulation also upregulated Bax and active-caspase 3 and downregulated PCNA and cyclinD1, suggesting that this lncRNA depletion increased the apoptosis rate among PCa cells after IR exposure. HULC knockdown also causes G0/G1 cell cycle arrest, while its overexpression arrests cells at the S phase. Additionally, HULC downregulation elevated phosphorylated levels of Beclin-1 and promoted autophagy through inhibition of the mTOR pathway [50]. 

Cancer susceptibility 19 (CASC19) lncRNA strongly contributes to the radioresistance of NPC cells by promoting autophagy through the AMPK/mTOR pathway. CASC19 is overexpressed in NPC cells and is associated with radioresistance. However, its knockdown enhances radiosensitivity and decreases autophagy via the AMPK/mTOR pathway. Inhibition of the studied lncRNA expression caused higher levels of IR-induced DNA damage, elevated IR-induced apoptosis with PARP1, and enhancement of cleaved caspase-3. This indicates that silencing CASC19 disturbed the protective effect of autophagy on IR-induced apoptosis. The abovementioned lncRNA contributes to the radioresistance of NPC cells by promoting autophagy and inhibiting apoptosis through the AMPK/mTOR signaling pathway [51].

### 2.3. Reactive Oxygen Species (ROS)

Ionizing radiation causes ROS generation and subsequently oxidative stress. Additionally, the process of tumor development itself generates significant amounts of these reactive molecules due to an increase in DNA mutations, high genome instability, and cell proliferation. Wang et al. determined that novel lncRNA AL033381.2 may exert its oncogenic function in hepatocellular carcinoma (HCC) through interacting with the PRKRA protein and targeting genes related to oxidative stress. Experiments carried out on mice with HCC xenografts treated with complexes combining nanoparticles and AL033381.2 siRNA showed anticancer effects leading to a reduction of tumor volume and weight. This lncRNA has the potential to become a therapeutic target in the future [52]. 

One of the factors promoting cancer-related oxidative stress is hypoxia—an oxygen deficiency resulting from intensive cell proliferation. This phenomenon is a characteristic feature of non-small cell lung cancer. It has been proven that lncRNA PVT1 regulates HIF1α by sponging miR-199a-5p in NSCLC cell lines. Moreover, it can be a potential therapeutic target supporting the fight against hypoxia and thus IR resistance [53].

Another lncRNA modulating HIF1α expression levels is HOTAIR. Studies carried out on CC cell lines indicated that high levels of this lncRNA were associated with developing resistance to radiotherapy. Interestingly, IR tends to diminish HOTAIR and HIF1α levels in cells, which results in decreasing cell viability and tumor growth, as well as inhibiting apoptosis. This implies that induced reduction of studied lncRNA expression accompanied by radiation treatment could bring satisfactory results in CC therapy [54].

### 2.4. DNA Damages and Repair Mechanisms

Radiotherapy induces cell death predominantly via promoting DNA double-strand breaks (DSBs).

A study by Wang et al. indicated that lncRNA LINC01134 is significantly overexpressed in HCC and affects the response to IR by regulating DNA damage repair mechanisms. This molecule acts as a molecular sponge sequestering miR-342-3p and recruiting insulin-like growth factor 2 mRNA binding protein 2 (IGF2BP2) to modulate mitogen-activated protein Kinase 1 (MAPK1) expression. LINC01134 depletion resulted in a higher DNA damage rate, tumor growth inhibition, and radioresistance reduction in HCC cells [55]. 

Another lncRNA playing a crucial role in modulating DNA repair mechanisms is LINC-PINT. Experiments carried out on NPC cells showed that this lncRNA might be a radiosensitizing tumor suppressor reducing these types of molecular pathways through the ATM/ATR-Chk1/Chk2 signaling axis. Additionally, LINC-PINT interacts with DNA-dependent kinase proteins (DNA-PKcs), inhibiting DNA damage response cascades and mediating cell cycle arrest predominantly in the G2 phase, in which the cell is the most vulnerable to IR [56]. 

LINC00312 is significantly downregulated in NPC and correlates with poor radiotherapy efficacy. On the other hand, LINC00312 overexpression results in decreased cell viability, impaired colony formation ability, cell cycle arrest in the G0/G1 phase, and better overall survival of NPC patients. Its overexpression enhances the sensitivity of xenograft tumors to radiation *in vitro*.

Studied lncRNA contributes to the NPC cells’ radioresistance through impairing DNA damage repair. LINC00312 inhibits the recruitment of DNA-PKc to Ku80 in response to DNA double-strand breaks. Overexpression of LINC00312 combined with exposure to irradiation decreased levels of p-ATM, p-ATR, p-Chk1, and p-Chk2, which play a crucial role in DNA damage checkpoint control and tumor inhibition. All of these results show that LINC00312 affects radiosensitivity by regulating the DNA damage repair pathway [57].

Yao et al. observed that the ANKHD1/MALAT1/YAP1 feedback loop induces radioresistance of CRC cells through regulating processes of DNA-damage repair, probably via the YAP1/AKT axis. They have proven that the knockdown of ANKHD1 and/or lncRNA MALAT1 significantly improves radiotherapy efficacy [58].

A study by Feng et al. described the STAT1/LINC00504/CPEB2/TAF15 interaction axis, which modulates the BC cells’ response to radiotherapy. The examined lncRNA could affect DNA damage through its influence on ATM/ATR activation. LINC00504 depletion weakened the survival rate of BC cells, which was further enhanced by applied IR, suggesting its therapeutic potential [59]. Moreover, Wang et al. discovered different lncRNA, whose downregulation could significantly improve BC patients’ response to radiotherapy. LINC02582 combines with ubiquitin-specific peptidase 7 (USP7) to deubiquitinate and stabilize checkpoint kinase 1 (CHK1), affecting DNA damage response, which in consequence leads to radioresistance. It has been proven that miR-200c, a known radiosensitizer of BC, directly regulates this lncRNA expression. Induced overexpression of said miRNA suppressed DNA repair mechanisms and elevated γ-H2AX levels after IR. Survival analysis corroborated the favorable impact of high miR-200c expression levels on patients’ outcomes, implying the importance of further studying the LINC02582 mechanism of action and its possible future application in treatment [60].

HOTAIR is significantly upregulated in many cancer types, including BC tissue. Its overexpression is strictly correlated with cells’ radioresistance and shows a poor prognosis for patients. Silencing HOTAIR caused significantly reduced cell proliferation and colony formation ability [61,62]. Additionally, HOTAIR upregulates HSPA1A, a well-known stress-inducible oncogene in irradiated BC cells. On the other hand, the examined lncRNA is negatively correlated with miR-449b-5p, which has the ability to reverse all effects of HOTAIR and HSPA1A. Taking this together, HOTAIR is capable of enhancing the growth of BC tumors under IR-induced stress through the miR-449b-5p/HSPA1A signaling pathway [61]. According to the results from another study considering HOTAIR and BC, alongside overexpressed HOTAIR, expression of DNA damage repair factors, such as Ku70, Ku80, DNA-PKcs, and ATM, was significantly increased. Additionally, higher HOTAIR expression induced recruitment of EHZ2 to the specific target gene c-MYC. In addition, the above lncRNA knockdown in breast cancer cells also resulted in a decrease in the number of cells in phase S, causing a significant increase in apoptosis [62].

In the triple-negative BC (TNBC), the process of DSB repair is regulated by lncRNA in the non-homologous end joining (NHEJ) pathway 1 (LINP1). This lncRNA plays the role of a scaffold that promotes interaction between Ku80 and DNA-PKcs, modulating the non-homologous end joining (NHEJ) pathway. Interestingly, Zhang et al. have also indicated that LINP1 expression could be regulated by EGF signaling, and in particular its activation of the RAS-MEK-JNK pathway, as well as the p53 pathway, which is mediated by the miR-29. Depletion of the studied lncRNA expression level might diminish DSB repair activity and lead to tumor radiosensitization [63]. LINP1 has a significant impact not only on TNBC biology but also affects CC development. In this type of cancer, this lncRNA also augments radioresistance via modulating the NHEJ pathway. Wang et al. determined that LINP1 undergoes IR-induced translocation from cytosol to the nucleus, where it interacts with the aforementioned proteins—Ku80 and PKcs. LINP1 silencing with subsequent IR leads to delayed DNA repair mechanisms, promoted cell apoptosis, and increased radiosensitivity in CC cell lines [64]. The above suggests promising therapeutic or prognostic potential of the said lncRNA [63,64].

Analyses carried out on glioma cell lines indicated that lncRNA SNHG18 plays a notable role in modulating cancer response to radiotherapy. Its depletion caused increased γ-H2AX and cleaved caspase-3 levels, affecting the DNA damage response. Additionally, SNHG18 expression was negatively correlated with semaphorin 5a (Sema5A) levels, which potentiated radioresistance. These results seem to underline the importance of further SNHG18-related studies [65].

Dynamin 3 opposite strand (DNM3OS) is highly expressed in ESCC cell lines as well as in tumor tissue compared to normal, matched tissues. The decreased expression of DNM3OS causes significantly increased radiosensitivity *in vitro* and *in vivo*. This lncRNA regulates cellular DNA damage response in ESCC. Its inhibition enhances irradiation-induced DNA damage and attenuates DNA repair response. The downregulation of the studied lncRNA resulted in elevated expression of the DNA damage markers γH2AX and PARP and decreased expression of DNA repair proteins such as p-ATM, Rad50, p-CHK2, Ku80, MRE11, NBS1, DNA-PKcs, and p53 in response to irradiation. Interestingly, cancer-associated fibroblasts (CAFs) promote DNM3OS expression in a PDGFβ/PDGFRβ/FOXO1 signaling pathway-dependent manner in ESCC, resulting in enhancing radioresistance [66].

The lncRNA CYTOR is highly correlated with the radioresistance of NSCLC cells. CYTOR is significantly overexpressed in NSCLC cell lines and tissues and is responsible for a poor prognosis of patients. Silencing the said lncRNA enhances radiosensitivity in NSCLC cells, weakening colony formation ability and expressing high expression of γH2AX. CYTOR knockdown also enhances the radiosensitivity of xenograft tumors in mice. The studied lncRNA also sponges miR-206, which can reverse all CYTOR effects on cells. However, miR-206 regulates PTMA, which has a similar impact on NSCLC cells as CYTOR, enhancing the radioresistance of NSCLC cells [67]. 

### 2.5. Changes in Cellular Phenotype

A study by Zhu et al. discovered that lncRNA RBM5-AS1 is upregulated in radioresistant medulloblastoma (MB) cell lines and implements its oncogenic function through stabilizing the sirtuin 6 (SIRT6) protein. This interaction has been associated with the self-renewal of medulloblastoma cancer stem cells (CSCs). Knockdown inhibited viability, tumor growth, and expression of a known stemness marker—CD133, simultaneously improving IR efficacy through promoting DNA damage and apoptosis [68]. A different lncRNA linked with modulating CSC phenotypes and activity is MALAT1. Experiments carried out on NPC cell lines determined that the MALAT1/miR-1/slug axis affects cancer radioresistance. Additionally, depletion of the said lncRNA inhibited cell proliferation and invasion, which emphasizes its therapeutic potential [69].

Moreover, Lin et al. reported that lncRNA NEAT1 regulates the phenotype of the TNBC cells through interaction with NAD(P)H:quinone oxidoreductase 1 (NQO1). Induced reduction in lncRNA expression level potentiated radiosensitivity, inhibited proliferative ability and CSC activity, and diminished expression levels of known stemness genes—BMI1, Oct4, and Sox2. These results suggest that NOQ1 bioactivatable compounds could be used as a potential therapeutic target inhibiting radioresistance [70]. 

Ye et al. described a complex interaction axis consisting of FEZF1-AS1/miR-107/ZNF312B, which promotes the progression of pancreatic ductal adenocarcinoma (PDAC). In addition, it facilitates in cancer cells the Warburg effect—a unique metabolic phenotype with increased glycolysis and reduced oxidative phosphorylation despite oxygen availability. Downregulation of lncRNA FEZF1-AS1 caused inhibition of tumor growth, probably through modulation of the apoptosis and in the G1-S checkpoint [71]. The discovered regulatory network provided an interesting new strategy that could help reduce radioresistance, which, as implied by Liu et al., can be mediated by glycolysis [72]. During their studies on cutaneous malignant melanoma (CMM), the authors presented the LINC00518/miR-33a-3p/HIF1α negative feedback loop, which caused metabolic changes and promoted cells’ malignant phenotypes, consequently decreasing radiotherapy effectiveness [72].

A recent study by Yi et al. identified lncRNA PTPRG-AS1/miR-194-3p/PRC1 regulatory circuitry that modulates radiosensitivity and metastatic phenotype of NPC cell lines. Knockdown of lncRNA or induced overexpression of miRNA prevented cell migration, invasion, and growth, improving response to the treatment [73]. Likewise, a type of lncRNA called CYTOR in NSCLC acts as a molecular sponge binding miR-195 and modulating tumors’ malignant phenotypes. Silencing of this lncRNA diminished proliferation, migration, and invasion of cancer cells, leading to enhancement of IR-induced therapeutic effects [74]. Another lncRNA affecting tumors’ metastatic ability is HOTAIR. Yang et al. discovered that its knockdown in CRC cell lines reduces proliferation and subsequently inhibits cell invasion, as well as promoting apoptosis in an IR-mediated manner [75]. Furthermore, in CC cell lines, this lncRNA is also upregulated and exerts an oncogenic function. HOTAIR enhanced cell migration, invasion, and proliferation, by reducing p21 protein levels. Moreover, its high expression causes suppression of apoptosis [76]. The HOTAIR’s significant influence on the development of a metastatic phenotype implies its therapeutic potential [75,76].

## 3. Potential Use of lncRNAs as Biomarkers

Zhang et al. identified a three-lncRNA-based signature that showed significant prognostic accuracy in ESCC patients who had undergone neoadjuvant chemoradiotherapy (nCRT). This molecular model consisting of SCAT1, PRKAG2-AS1, and FLG-AS1 can accurately predict which patients will benefit from nCRT and obtain a complete pathological response. Such a signature is suitable for long-term treatment evaluation and may be the first step to individualized therapy of ESCC [77]. A study by Li et al. indicated that highly expressed lncRNA Rpph1 is a promising diagnostic biomarker of esophageal carcinoma. Its level has been associated with T stage, N stage, and clinical stage, as well as prognosis of patients’s outcome. Experiments carried out on cell lines proved that Rpph1 silencing can suppress tumor development and enhance radiosensitivity, underlining the tremendous potential of this molecule [78]. Many studies indicated that different lncRNA molecules have the potential ability to be applied as biomarkers in oncology and personalized radiotherapy. 

A good biomarker should be simply obtained from diverse sources, it should be of high quantity and quality and the measurement methods should not be complicated. lncRNAs may be found in various sources such as tissues, cell lines, peripheral blood, serum, plasma, saliva, urine, exosomes, and FFPET [16,79,80,81,82,83,84]. However, not all are present in every type of biological material. For instance, MALAT1, MEG3, HULC, HOTAIR, UCA1, and NEAT1 were all previously found in OSCC tissues but in the saliva of the same patients, only MALAT1 and HOTAIR were detectable [16,83]. lncRNAs are also easily extracted from these samples; despite the lack of dedicated commercial kits for lncRNAs extraction, it can be done with a standard TRIzol method or ready-to-use commercial kits for RNA extraction. There is a lack of standardized extraction methods for lncRNAs, as well as storage recommendations or even reference genes for qRT-PCR [16]. However, some lncRNAs are more stable than miRNAs. Their half-life is approximately 16 h and they are resistant to RNase A digestion and room temperature incubation [16,84]. Most lncRNAs are present at low copy numbers, but the addition of polyA tails and annealing anchor dT adapters before cDNA synthesis solves the problem. This solution also enhances the specificity and sensitivity of lncRNA quantification [16,85]. Additionally, lncRNAs may be examined using many different methods such as immunoprecipitation, in situ hybridization, Au-NP assay (gold nanoparticle-based), Northern blot, high resolution melting (HRM), microarrays, next generation sequencing (NGS), and PCR methods (real-time PCR, droplet PCR) [16,79,80,86,87,88]. The choice of the procedure depends on the study (screening or specific detection), type of material, and costs [16]. lncRNAs show properties of good biomarkers for being easy to obtain, specific, and easy to measure. Even small challenges in working with lncRNAs are simple to overcome, making them very promising biomarkers.

Finding the appropriate biomarker of radiosensitivity/radioresistance or a molecule that modulates the response to this type of treatment may improve the process of selecting an accurate irradiation regimen. This type of personalized approach might significantly reduce the adverse effects associated with IR. It is well known that complications such as fibrosis in surrounding normal tissues, immune toxicity, and inflammation can substantially decrease the quality of patients’ life [89,90]. Designing a panel of biomarkers dedicated to individual cancers would provide the opportunity to propose to individuals sensitive to radiation a therapy that is less intensive than the standard treatment scheme. On the other hand, people with radiation-resistant tumors could benefit from a higher IR dose, more frequent applications, or, e.g., linear energy transfer (LET) radiation [91]. This type of therapy effectively eradicates cancer with the lowest biologically effective dose and the maximum reduction in toxicity. A study by Niemantsverdriet et al. proved that cell lines treated with high-LET radiation had significantly lower survival and higher apoptosis rates [91]. The method is not generally available because it is more expensive and complex than conventional therapy. The use of specific biomarkers would enable the process of qualifying patients for treatment strategies that will be the most beneficial.

**Figure 2:**
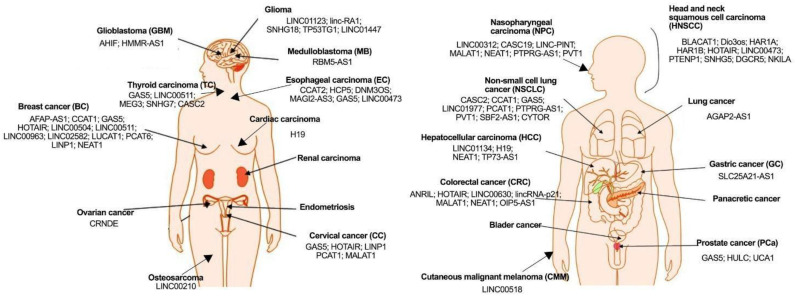
lncRNAs with diagnostic potential in personalized radiotherapy in different types of human cancers.

Examples of known lncRNAs with diagnostic potential in cancers are summarized in Figure 2 and Table 1.

**Table 1:** Known lncRNAs affecting cell radiotherapy response.

Gene Name	Cancer Type	Expression	Impact on Radiotherapy	Targets	Reference
AFAP-AS1	Breast cancer (BC)	Upregulated	AFAP-AS1 overexpression enhances radioresistance of BC cells (promoted cell proliferation, invasion, tumor growth, inhibits apoptosis)	Wnt/β-catenin	[92]
AGAP2-AS1	Lung cancer	Upregulated	Promotes the immunologic function after IR	miR-296/NOTCH2	[93]
AHIF	Glioblastoma (GBM)	Upregulated	AHIF knockdown enhances radiosensitivity	Bax/Bcl-2	[94]
ANRIL	Colon cancer	Upregulated	ANRIL suppress radiosensitivity by binding to miR-181a-5p and reversing functions of chitooligosaccharides (COS)	miR-181a-5p/ chitooligosaccharides (COS)	[44]
BLACAT1	Head and neck squamous cell carcinoma (HNSCC)	Upregulated	BLACAT1 knockdown enhances radiosensitivity of HNSCC cells	PSEN1	[95]
CASC19	Nasopharyngeal carcinoma (NPC)	Upregulated	CASC19 contributes to the radioresistance of NPC cells by promotion of autophagy and inhibition of apoptosis through AMPK/mTOR signaling pathway	PARP1/AMPK/mTOR	[51]
CASC2	Non-small cell lung cancer (NSCLC)	Downregulated	CASC2 overexpression induces radiosensitivity of NSCLC cells	PERK/CHOP	[96]
	Papillary Thyroid Cancer (PTC)	Downregulated	Low expression of CASC2 causes high IR resistance of PTC cells; overexpression of CASC2 results in higher IR sensitivity of PTC cells (induced cell viability and inhibited post-IR apoptosis); CASC2 enhances radiosensitivity in PTC by sponging miR-155	miR-155	[97]
CCAT1	Breast cancer (BC)	Upregulated	Downregulation of CCAT1 enhances radiosensitivity through miR148b negative regulation (decreased colony formation rate, promoted apoptosis)	miR-148b/miR-218/ZFX	[92,98]
	Non-small cell lung cancer (NSCLC)	Upregulated	Higher CCAT1 expression correlates with higher radioresistance of NSCLC cells; downregulation of CCAT1 can improve the radiosensitivity of NSCLC cells by mediating cell cycle arrest, DNA damage, and apoptosis	MAPK/MAPK1/ERK/ MEK	[33]
CCAT2	Esophageal carcinoma (EC)	Upregulated	CCAT2 knockdown results in radiosensitivity enhancement of EC cells (induced apoptosis); overexpressed CCAT2 causes EC cells to gain radioresistant features through inhibiting apoptosis via miR-145/p70S6K1 signaling pathways and by activating the Akt/ERK/p70S6K1 signaling pathways	miR-145/p70S6K1/p53/ c-Myc/Akt signaling pathway	[99]
CRNDE	Ovarian cancer	Upregulated	CRNDE silencing resulted in enhanced radiosensitivity, inhibited clone formation and tumor growth in mice	No data	[28]
CYTOR	Non-small cell lung cancer (NSCLC)	Upregulated	Silencing CYTOR results in enhanced radiosensitivity of NSCLC cells (weak colony formation, high levels of H2AX); CYTOR binds to miR-206, silencing it and causing upregulation of PTMA, resulting in radioresistance	miR-206/PTMA	[67]
			Suppresses radiosensitivity through regulating malignant phenotypes	miR-195	[74]
DGCR5	Laryngeal carcinoma (LC)	Upregulated	Knockdown could sensitize tumor cells to radiation through modulating miR-195	miR-195	[37]
Dio3os	Head and neck squamous cell carcinoma (HNSCC)	Downregulated after IR	No data	MYH7B/SRCAP/ HELZ2/NOS1/CROCC/CEP250/LPP/ABI2/ HERC2/RTEL1/ SMC1A1/HERC1/GAS7/NOTCH2/PKD1/ CFLAR/FAT3/FAT2/ CELSR3/CBL/NCOR2	[100]
DNM3OS	Esophageal squamous cell carcinoma (ESCC)	Upregulated	Decreased DNM3OS cause significantly increased radiosensitivity *in vitro* and *in vivo*	Cancer associated fibroblasts (CAFs)/PDGFB/ PDGFRB/FOXO1	[66]
GAS5	Thyroid carcinoma (TC)	Downregulated	GAS5 overexpression enhances radiosensitivity	miR-362-5p/SMG1	[101]
	Cervical cancer (CC)	Downregulated	GAS5 overexpression enhances CC cells radiosensitivity	miR-106b/IER3	[102]
	Breast cancer (BC)	Downregulated after IR further decrease in expression level	Induced overexpression reduces miR-21 expression leading to radiosensitization	miR-21	[31]
	Esophageal squamous cell carcinoma (ESCC)	Downregulated	Simultaneous upregulation with miR-21 knockdown improves radiosensitivity after IR	miR-21/RECK	[41]
	Prostate cancer (PCa)	Downregulated	Artificially elevated level enhances α-solanine-induced radiosensitivity	miR18a	[30]
	Non-small cell lung cancer (NSCLC)	Downregulated	Enhances radiosensitivity	miR-135b	[103]
H19	Cardiac carcinoma	Upregulated	H19 knockdown resulted in enhanced radiosensitivity of cardiac carcinoma cells	miR-130a-3p/miR-17-5p	[104]
	Hepatocellular carcinoma (HCC)	Downregulated	H19 overexpression enhances radiosensitivity of HCC cells through the miR-193a-3p/PSEN1 axis (promoted apoptosis, inhibited DNA double-strand break repair)	miR-193a-3p/PSEN1	[105]
HAR1A	Head and neck squamous cell carcinoma (HNSCC)	Downregulated after IR	No data	RANBP2/LPP/ABI2/ HELZ/PHC3/HERC1/ MTO5A/FZD3/ CTNNA3/CBL/BMPR2/FAT3/CFLAR	[100]
HAR1B	Head and neck squamous cell carcinoma (HNSCC)	Downregulated after IR	No data	LPP/ABI2/RSF1/HERC2/HELZ/FZD3/CFLAR/ FAT3/FRK/FER/PDK1/ FAT1	[100]
HCP5	Esophageal carcinoma (EC)	Upregulated	Knockdown of HCP5 enhances radiosensitivity trough modulating the Akt signaling pathway	miR-216a-3p/PDK1	[26]
HMMR-AS1	Glioblastoma (GBM)	Upregulated	Knockdown may suppress and radiosensitize the tumor	may regulate ERK1/2 by altering HMMR expression	[34]
HOTAIR	Colorectal cancer (CRC)	Upregulated; increases after IR in dose-dependent manner	Inhibits radiotherapy efficacy through regulating miR-93/ATG12-mediated autophagy	miR-93/ATG	[39]
	Colorectal cancer (CRC)	Upregulated	Downregulation of HOTAIR enhanced radiosensitivity via reducing cell proliferation and invasiveness	No data	[75]
	Head and neck squamous cell carcinoma (HNSCC)	Upregulated after IR	Higher expression of HOTAIR is correlated with a higher resistance to radiotherapy in colon and breast cancer cell lines; high expression of HOTAIR is connected with the EMT process, maintaining of cancer initiating cells, and aggressive types of HNSCC	LPP/ABI2/NOS1/ CFLAR/REL/FAT3/ PDK1/FZD3/SMAD2/ FRK/SRCAP/WNT2B/ CBL	[100]
	Cervical cancer (CC)	Upregulated	Leads to tumor radioresistance via increasing HIF1α expression	HIF1α	[54]
	Cervical cancer (CC)	Upregulated	Promotes radioresistance through p21 inhibition	p21	[76]
	Breast cancer (BC)	Upregulated	Downregulation of HOTAIR enhanced radiosensitivity	miR-218	[106]
	Breast cancer (BC)	Upregulated	Overexpression of HOTAIR results in higher radioresistance of BC cells; HOTAIR overexpression enhances the cell proliferation and growth under irradiation stress; HOTAIR knockdown resulted in increased apoptosis and a reduced number of BC cells in the S phase of a cell cycle; its expression is positively correlated with DNA damage repair factors	HSPA1A/ miR-449b-5p/EZH2/PRC2/EED/ SUZ12/Ku70/Ku80/ DNA-Pk/ATM	[61,62]
HULC	Prostate cancer (PCa)	Upregulated after IR	HULC knockdown enhances sensitivity of PCa cell to IR; cell apoptosis and proliferation induced by IR are enhanced by HULC knockdown and decreased by HULC overexpression	Bax/PCNA/cyclinD1/ caspase-3/Beclin-1/ p-4E-BP1	[50]
LINC00210	Osteosarcoma	Upregulated	Knockdown of LINC00210 results in enhanced radiosensitivity (after IR: decreased cell viability, induced apoptosis, inhibited levels of CyclinD1 and Bcl-2, increased levels of p21 and Bax); regulates radiosensitivity through the miR-342-3p/GFRA1 axis	miR-342-3p/GFRA1/ CyclinD1/Bcl-2/p21/Bax	[45]
LINC00312	Nasopharyngeal carcinoma	Downregulated	Overexpression suppresses radiotherapy resistance	RAD50/MRE11/NBS1/ Ku80	[57]
LINC00473	Esophageal squamous cell carcinoma (ESCC)	Upregulated	Reduces radiotherapy effectiveness increasing cancer proliferative ability	miR-497-5p/CDC25A, miR-374a-5p/SPIN1	[35,36]
	Head and neck squamous cell carcinoma (HNSCC)	Upregulated	LINC00473 knockdown enhances radiosensitivity of HNSCC cells	Bax/Bcl2/Wnt/β-catenin pathway	[107]
LINC00504	Breast cancer (BC)	Upregulated	Decreases cell radiosensitivity by regulating CPEB2 expression	TAF15/CPEB2	[59]
LINC00511	Thyroid carcinoma (TC)	Upregulated	Potentiates resistance to radiotherapy by modulating the TAF1/JAK2/STAT3 axis	TAF1/JAK2/STAT3	[108]
	Breast cancer (BC)	Upregulated	LINC00511 knockdown enhances radiosensitivity (restricts cell proliferation, promotes apoptosis, and inhibits tumor growth)	STXBP4/miR-185	[92]
LINC00518	Cutaneous malignant melanoma (CMM)	Upregulated	Potentiates radioresistance through enhancing glycolytic metabolism	miR-33a-3p/HIF1α/ LDHA	[98]
LINC00630	Colorectal cancer (CRC)	Upregulated	Silencing could increase radiosensitivity by epigenetically repress BEX1 expression	EZH2/BEX1	[38]
LINC00963	Breast cancer (BC)	Upregulated	Highly expressed LINC00963 causes BC cells to enhance radioresistance; its silencing results in an increase of radiosensitivity (restrains cell proliferation, impairs colony formation and tumor growth, arrest cells at the G0/G1 phase, stimulates apoptosis); LINC00963 induced radiosensitivity through the miR-324-3p/ACK1 axis	miR-324-3p/ACK1/ CDK6/p27/CyclinD1	[109]
LINC01123	Glioma	Upregulated	Enhances radioresistance by creating the LINC01123/miR-151a/CENPB axis	miR-151a/CENPB	[110]
LINC01134	Hepatocellular carcinoma (HC)	Upregulated	Augments resistance to radiotherapy via modulating the MAPK1 signaling pathway	miR-342-3p/IGF2BP2/ MAPK1	[55]
LINC01447	Low-grade Glioma	Upregulated	LINC01447 inhibition results in radiosensitivity enhancement in low-grade glioma cells (decreased cell viability, inhibited colony formation, increased apoptosis)	No data	[111]
LINC01977	Non-small cell lung cancer (NSCLC)	Upregulated	LINC01977 inhibition results in enhanced radiosensitivity in NSCLC cells (reduced colony formation, higher expression of H2AX)	No data	[112]
LINC02582	Breast cancer (BC)	Upregulated	Promotes radioresistance via the USP7/CHK1 signaling axis	USP7/CHK1	[60]
LINC-PINT	Nasopharyngeal carcinoma (NPC)	Downregulated	Artificial upregulation potentiates radiosensitivity through an increase in apoptosis rate	ATM/ATR-Chk1/Chk2 pathway and DNA-PKcs	[56]
linc-RA1	Glioma	Upregulated	Strengthens radioresistance via inhibiting autophagy activation	H2Bub1/USP44	[49]
lincRNA-p21	Colorectal cancer (CRC)	Downregulated IR cause further decrease in expression level	Enhances radiosensitivity after IR through activating pro-apoptotic mechanisms	Wnt/β-catenin/c-myc and cyclin D1 axis, Noxa	[40,113]
LINP1	Cervical cancer (CC)	Upregulated	Augments radioresistance via enhancing dsDNA break repair through the NHEJ pathway	Ku80, DNA-PKcs	[64]
	Triple negative breast cancer (TNBC)				[63]
LUCAT1	Breast cancer (BC)	Upregulated	LUCAT1 knockdown results in enhanced radiosensitivity of BC cells through the miR-181a-5p/KLF6/KLF15 axis (reduced cell proliferation, migration, viability, and invasion)	miR-181a-5p/KLF6/ KLF15	[114]
MAGI2-AS3	Esophageal squamous cell carcinoma (ESCC)	Downregulated	MAGI2-AS3 silencing strengthens resistance of ESCC cells to IR *in vivo*	HOXB7/EZH2/ miR-374b-5p/ CCDC19/miR-15b-5p	[43]
MALAT1	Colorectal cancer (CRC)	Upregulated	Silencing may increase radiosensitivity through the YAP1/AKT axis	ANKHD1/YAP1/AKT	[58]
	Nasopharyngeal carcinoma (NPC)		Downregulation strengthens IR effects	miR-1/slug	[69]
	High-risk HPV-positive cervical cancer (HR-HPV+ CC)		Increases radioresistance through negatively regulating miR-145	miR-145	[32]
MEG3	Thyroid carcinoma (TC)	Downregulated	MEG3 overexpression results in higher TC cells radiosensitivity (inhibited proliferation, promoted apoptosis, and DNA damage) through miR-182 sponging	miR-182	[115]
NEAT1	Colorectal cancer (CRC)	Upregulated; increases after IR in time-dependent manner	Augments radioresistance by promoting IR-induced pyroptosis	miR-448/GSDME	[46]
	Triple negative breast cancer (TNBC)	Upregulated	Knockdown improves cell sensitivity to radiation via positive regulation of NQO1	NQO1/miR-218	[70,92]
	Nasopharyngeal carcinoma (NPC)	Upregulated	NEAT1 downregulation sensitizes NPC cells to radiation	miR-204/ZEB1	[116]
	Hepatocellular carcinoma (HCC)	Upregulated	NEAT1_2 down-regulation enhances radiosensitivity of HCC cells	miR-101-3p/WEE1	[117]
NKILA	Laryngeal carcinoma (LC)	Downregulated	Overexpression of NKILA reduces radioresistance of LC cells by inhibiting p65 nuclear translocation (suppresses cell viability, DNA synthesis capability, and migration ability)	p65	[118]
OIP5-AS1	Colorectal cancer (CRC)	Downregulated	Overexpressed OIP5-AS1 impedes cell viability, promotes radio-induced apoptosis, and enhances radiosensitivity of CRC cells through the miR-369-3p/DYRK1A axis	miR-369-3p/DYRK1A	[119]
PCAT1	Non-small cell lung cancer (NSCLC)	Upregulated	Inhibition of PCAT1/SOX2 together with radiation promotes IR-induced anti-tumor immune responses	SOX2/cGAS/STING	[120]
	Cervical cancer (CC)	Upregulated	PCAT1 knockdown enhances radiosensitivity of CC cells through regulating the miR-128/GOLM1 axis (inhibited cell proliferation, migration, and invasion)	miR-128/GOLM1	[121]
PCAT6	Breast cancer (BC)	Upregulated	PCAT6 knockdown results in enhanced radiosensitivity (reduced cell proliferation, promoted apoptosis)	TPD52/miR-185-5p	[92]
PTPRG-AS1	Non-small cell lung cancer (NSCLC)	Upregulated	Diminishes radiotherapy efficacy by modulating miR-200c-3p/TCF4	miR-200c-3p/TCF4	[122]
	Nasopharyngeal carcinoma (NPC)		Silencing leads to significant improvement of radiosensitivity	miR-194-3p/PRC1	[73]
PTENP1	Head and neck squamous cell carcinoma (HNSCC)	Upregulated after IR	Inhibition of miR-21 causes cell radiosensitivity by increasing the PTEN protein expression in HNSCC	miR-21	[100]
PVT1	Non-small cell lung cancer (NSCLC)	Upregulated; the highest levels reached under hypoxia	Induced downregulation could weaken radioresistance by reducing hypoxia	HIF1α/miR-199a-5p	[53]
	Nasopharyngeal carcinoma (NPC)	Upregulated	Induces radioresistance; knockdown of PVT1 enhances the radiosensitivity of NPC cell lines	No data	[42]
RBM5-AS1	Medulloblastoma (MB)	Upregulated; the highest levels in radioresistant medulloblastoma cells	Promotes radioresistance and cancer stemness	SIRT6	[68]
SBF2-AS1	Non-small cell lung cancer (NSCLC)	Upregulated	Downregulation of SBF2-AS1 enhances NSCLC cells’ radiosensitivity through the miR-302a/MBNL3 axis (inhibited cell proliferation, enhanced apoptosis, reduced tumor growth in mice)	miR-302a/MBNL3	[123]
SLC25A21-AS1	Gastric cancer (GC)	Downregulated	Overexpression of SLC25A21-AS1 enhances the radiosensitivity and inhibits the malignant behaviors of GC cells by upregulating the miR-15a-5p/SNCG axis	miR-15a-5p/SNCG	[124]
SNHG5	Head and neck squamous cell carcinoma (HNSCC)	Downregulated after IR	No data	LPP/ABI2/HELZ/ RANBP2/CEP250/ CDK6/HERC1/PHC3/ MYO5A/FZD3/CFLAR/FAT3/FAT1/FRK/ SMAD2/BMPR2/PTEN/ZMAT3/MDM4/FAT2/APC/FER	[100]
SNHG7	Thyroid carcinoma (TC)	Upregulated	Induced depletion may diminish radioactive iodine resistance through the PI3K/Akt pathway	miR-9-5p/DPP4/PI3K/ Akt	[25]
SNHG18	Glioma	Upregulated	Potentiates radioresistance via modulating levels of DNA damage response proteins	Sema5A	[65]
TP53TG1	Glioma	Upregulated	Induced downregulation could lead to radiation-mediated cancer growth inhibition	miR-524-5p/RAB5A	[48]
TP73-AS1	Hepatocellular carcinoma (HCC)	Upregulated	TP73-AS1 knockdown results in radiosensitivity enhancement of HCC cells through the PTEN/Akt signaling pathway (reduced proliferation, reduced colony formation ability, and induced apoptosis)	PTEN	[125]
TTN-AS1	Large intestine cancer	Upregulated	TTN-AS1 knockdown resulted in radiosensitivity enhancement in large intestine cancer cells	miR-134-5p/PAK3	[126]
UCA1	Prostate cancer (PCa)	Upregulated	UCA1 knockdown enhances radiosensitivity of PCa cells	miR-331-3p/EIF4G1	[127]
XIST	Neuroblastoma (NB)	Upregulated	Higher XIST expression is correlated with higher NB cells’ radioresistance; its silencing results in inhibition of cell cycle progression, cell proliferation, colony formation, and enhanced post-IR apoptosis rate; it regulates IT through the miR-375/L1CAM axis	miR-375/L1CAM	[128]

## 4. Conclusions and Future Directions

Over time, long non-coding RNAs, despite their lack of protein-coding ability, have proven to be essential molecules in carcinogenesis but also the subsequent functioning of cancer cells. Their expression directly affects mRNAs, miRNAs, and other lncRNAs. Moreover, they have the ability to regulate transcription factors and chromatin modifications, as well as influence the chromatin condensation state. Due to little knowledge about them and the continuous analysis of their activity in particular cell types, the molecular mechanisms of tumorigenesis are still being explored. However, there are hopes that they will be important diagnostic tools in the future, including in assessing the success of radiotherapy, which is the most common treatment method among cancer patients. lncRNAs regulate the crucial processes implicated in radioresistance/radiosensitivity. It is known that these molecules are implicated in DNA damage response and repair mechanisms, regulation of HIF1α and ROS levels, mediation of cell death, autophagy, cell cycle, and proliferation. Many reports indicated potential uses of lncRNAs in the personalization of radiotherapy, but these studies were carried out on small groups of patients and were not randomized. A great opportunity is seen in the analysis of data from the Cancer Genome Atlas (TCGA) project and the use of AI. Big data analysis gives the possibility of selecting proper lncRNAs, which can be used for validation based on a larger group of patients. This approach potentially will give the ability to clinically use lncRNA-based panels for personalization of radiotherapy.

## Data Availability

All data are available online with common access.
